# CompPhish: A comprehensive dataset for diversified phishing detection

**DOI:** 10.1016/j.dib.2026.113219

**Published:** 2026-09-03

**Authors:** Richa Goenka, Meenu Chawla, Namita Tiwari

**Affiliations:** Department of Computer Science & Engineering, MANIT, Bhopal, India

**Keywords:** Phishing dataset, URL-based, HTML source code, Compromised domains, Brand-jacking, Machine learning

## Abstract

Most of the cyber attacks are initiated through phishing URLs, which are shared with the victims through multiple media. In spite of the research community proposing varied solutions, the volume and nature of such attacks have evolved unpredictably. To develop effective solutions, researchers require comprehensive datasets that encompass a wide range of attack types rather than focusing on a narrow subset. We present CompPhish, a processed dataset, comprising of 15,358 URLs paired with their respective HTML sources. 7204 URLs are phishing, and 8154 are legitimate. A set of 70 features, specifically curated to capture the properties exhibited by phishing URLs, is extracted. These features represent a diverse range of phishing attacks, including generic URL-based phishing attacks, phishing through brand-jacking, phishing sites hosted on compromised domains, and auto-downloadable malicious files embedded in webpages. Phishing URLs are gathered from PhishTank and OpenPhish, while legitimate URLs are compiled from multiple independent sources, like DataforSEO and GitHub.

By publishing the raw URLs and HTML codes along with the feature vectors, CompPhish attempts to aid the researchers in devising generalized, robust, and reliable machine learning based solutions for real-world phishing attempts.

Specifications Table

**Table:** 

Subject	Computer Sciences
Specific subject area	Computer Networks and Communications, Information Systems and Management, Artificial Intelligence.
Type of data	Excel file *(.xlsx),* HTML Source code *(.txt)*
Data collection	URLs: Public repositories like PhishTank and OpenPhish are used to collect the latest reported and verified Phishing URLs. Legitimate URLs are compiled from the DataForSEO Top-1000 websites list, covering widely used global domains. Additionally, manually verified safe URLs from GitHub, personal blogs and small business sites are included to ensure diversity. URL_HTML_Mapping: An excel file*(Mapping_file.xlsx*) is created with the column headers as *serial_number, url, label.* Python programming language is used to fetch the HTML content of each URL, which is saved locally and named according to its *serial_number*. URLs whose HTML source code could not be downloaded due to their inactive status are discarded.
Parameters for data-collection	• Only the verified phishing websites are included. • Legitimate websites are chosen from public community datasets. • Since the phishing websites are active for a very short duration, recently reported phishing URLs are included so that their HTML code can be downloaded.
Data Collection Period	September 2024 to August 2025
Description of Data	The processed dataset file (*All_features_threshold90.xlsx*) comprises 70 features, out of which, 47 features are extracted from the URL, while 23 features are extracted from the HTML content.
Class Composition	Phishing : 7204 (46.9%), Legitimate: 8154 (53.1%)
Data source location	Worldwide
Data accessibility	Repository: Mendeley Repository [1] URL: https://data.mendeley.com/datasets/fmbs4kp9wz/4
Related research article	None

## 1 Value of the Data

• This dataset consists of verified phishing and legitimate URLs along with their respective HTML source codes. Each URL is labelled as phishing or legitimate and is mapped to its HTML source code with a serial_number.
• For each URL, a set of 70 features are extracted, which are based on the traits indicated by multiple categories of phishing attacks, unlike the current available datasets. These features can indicate phishing attempts arising from domain squatting, URL obfuscation, malicious executable files, compromised domains, as well as lexical or structural manipulations in a URL.
• The researchers can utilize the dataset for a wide range of phishing detection tasks, including training and testing machine learning, deep learning, and hybrid detection models, feature selection, model benchmarking, comparative performance evaluation, and cross-validation studies. It can also serve as a benchmark for comparing newly developed phishing detection techniques under consistent experimental settings.
• New hand-crafted features can be extracted from the raw URLs and HTML content to facilitate the development of novel detection methodologies. This makes the dataset reproducible and a valuable asset for feature engineering.
• The dataset can be analyzed by the security professionals to study the evolving nature of phishing tactics, comparing URL-based and HTML-based models, feature importance and explainability studies, adversarial robustness evaluation, phishing trend analysis, and the development and validation of novel phishing detection algorithms.

## 2 Background

Phishing is a security risk that exploits trust and technical vulnerabilities to gain unauthorized access to sensitive information of the victims. The enhanced reliance on the internet for performing daily tasks is contributing to the rapid rise in phishing, advocating the need for the development of effective prevention and detection strategies. This is possible only if researchers have access to high-quality, comprehensive, and well-structured datasets.

Most of the public phishing datasets suffer from significant limitations. The dataset shared by the authors in [2] provides raw URLs along with their corresponding class labels. The absence of pre-extracted features requires the development of feature extraction pipelines before conducting any analysis. This process can be time-consuming, especially for those researchers who primarily focus on machine learning and deep learning techniques. A similar problem is observed in the dataset shared in [3], where the raw URLs and HTML source codes are provided, minus the phishing-related attributes.

The datasets published in [4,5], and [6] share only the features, and not the raw URLs or HTML source codes. Their major limitation is that they restrict reproducibility and verification of research findings. Since the raw samples are unavailable, researchers cannot validate the correctness of the extracted features. This also makes it difficult to compare different detection approaches under identical conditions. Another significant limitation is the limited scope for extracting novel features, thus restricting its usefulness. With 58,000 legitimate samples and 30,647 phishing samples, class imbalance is another drawback of the dataset shared in [4].

A dataset that includes a rich set of URL-based, HTML-based, and derived features is shared by the authors of [7]. However, only the raw URLs are provided in the publicly shared repository without including their corresponding HTML source files. This limitation can significantly reduce the long-term utility of the dataset. Phishing websites are short-lived and often inactive shortly after being reported. Consequently, researchers may be unable to access the HTML content at a later date, restricting the extraction of novel features, reducing reproducibility, and hindering the development of advanced phishing detection approaches.

The dataset [8] provides raw URLs together with a predefined set of URL-based features and offers greater flexibility than feature-only datasets. Its major drawback lies in the absence of webpage content, limiting the scope of research that can be conducted using the dataset at a later date. Such kind of datasets fail for those advanced phishing attacks that deliberately evade URL-based detection mechanisms.

There is a lack of datasets that integrate multiple layers of information required for advanced analysis. To bridge this gap, we present a comprehensive phishing dataset that includes raw URLs along with corresponding HTML source code, allowing in-depth URL and content-based analysis. Additionally, we provide 70 extracted features that cover lexical and structural URL characteristics, brand-jacking indicators, and HTML content attributes capable of detecting phishing sites hosted on compromised domains (PSHCD), malicious hyperlinks, and auto-downloadable content. This integrated resource supports the development of traditional machine learning, deep learning, and hybrid detection models. While the raw URLs are obtained from publicly available sources, the contribution of this work lies in the collection of corresponding HTML source code files, dataset curation, and multi-faceted feature extraction. By releasing both raw and processed data, the dataset promotes reproducibility, facilitates comparative research, and encourages innovation in phishing detection methodologies. Table 1 presents a comparison of CompPhish with existing datasets that are discussed.

**Table 1:** Comparison of CompPhish with existing datasets.

Dataset	Size	No. of Features	Raw URLs shared?	Raw HTML shared?	URL based features shared?	Content based features shared?	Remarks
[2]	73,576	NA	Yes	No	No	No	No pre-extracted features shared
[3]	60,252	NA	Yes	Yes	No	No	No pre-extracted features shared
[4]	88,647	111	No	No	Yes	No	Class imbalance can lead to incorrect results and does not support reproducibility
[5]	15,364	79	No	No	Yes	No	Does not support reproducibility
[6]	11,054	30	No	No	Yes	No	Does not support reproducibility
[7]	101,218	17	Yes	No	Yes	No	Restricted coverage of the phishing attack spectrum
[8]	235,795	54	Yes	No	Yes	Yes	Restricted ability for novel feature extraction from inactive URLs
CompPhish	15,358	70	Yes	Yes	Yes	Yes	Balanced dataset with raw samples and pre-extracted features shared to support reproducibility and facilitate advanced research

## 3 Data Description

***Mapping_File.xlsx*** – A Microsoft (MS) Excel file that provides a mapping between each serial_number and its corresponding URL and binary label (1 - phishing, 0 - legitimate). The *serial_number* column serves as the primary reference for each sample*.*

**All_HTML**: Folder containing the raw HTML source codes of each webpage, which are named with a unique serial_number followed by *.txt* extension*.* For example*, 10001.*txt contains the HTML source code of the URL with serial_number *10001.*

***sample_brands_new.csv*:** A list of commonly impersonated brands, compiled from multiple publicly available threat intelligence reports, phishing trend analyses, and cybersecurity monitoring platforms [[9], [10], [11], [12], [13]]. These sources regularly publish statistics on the organizations most frequently abused by attackers in phishing campaigns. Additionally, some top brands from diverse sectors such as banking, education, travel, and logistics, etc., are included to facilitate a wider outreach. The collected brand names are consolidated into a list with 1997 records, with duplicate entries removed, and uniformity ensured by using a standard domain naming pattern.

Along with the frequently targeted brands, the list also contains their associated legitimate domains (also known as protected domains), including alternate TLD (Top Level Domain) registrations, and official domain variants. These domains are used during brand-jacking feature extraction to distinguish legitimate brand-owned domains from potential typosquatting attempts and reduce false positives.

**Fig. 1:**
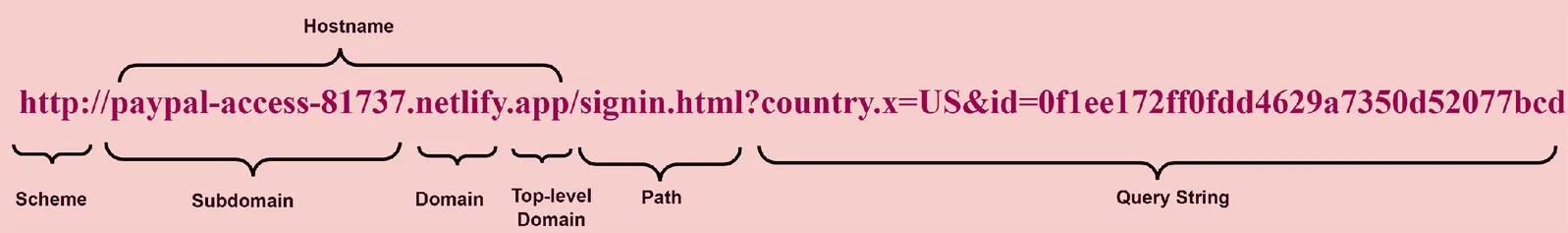
Components of a URL.

***All_Features_threshold90.xlsx****-* An MS Excel file containing 70 extracted features where each row corresponds to one web page and includes serial_number for mapping and label for classification. The features that are extracted are categorized as follows:

**1. Brand-jacking-based Features**: These features capture the phishing attempts that impersonate popular brands and deceive the users. The attackers register domain names that resemble the legitimate ones, with subtle variations that might be ignored by an unsuspecting individual. These features are extracted by using fuzzy string matching to compare the various components of the URL (Fig. 1) with the most vulnerable brands that are listed in *sample_brands_new.csv*. These features are presented in Table 2.

**Table 2:** Feature description: Brand-Jacking-based features.

S.No	Feature	Type of data	Description
F_1_	brand_subdomain	Boolean (0/1)	Presence of a brand in the subdomain
F_2_	brand_substring_domain	Boolean (0/1)	Presence of a brand as substring in the domain
F_3_	brand_typo_dom	Boolean (0/1)	Presence of a misspelt brand in the domain
F_4_	fake_tld	Boolean (0/1)	Presence of a brand with fake top-level domain
F_5_	brand_in_path_query	Boolean (0/1)	Presence of a brand in the path or query

**2. Generic URL-based lexical and structural features**: Described in Table 3, these features are derived from the syntactic and structural characteristics of a URL, which the attackers exploit to create deceptive links that appear genuine at first glance but contain anomalies.

**Table 3:** Feature description: URL-based lexical and structural features.

S. No	Feature	Type of data	Description
F₆	dots_path	Numerical (count-based)	Count of dots in the path of URL
F₇	url_more_than_mean	Boolean (0/1)	URL length greater than mean length of phishing URLs
F₈	domain_more_than_mean	Boolean (0/1)	Domain length greater than mean length of phishing domains
F₉	gibberish	Boolean (0/1)	Presence of a nonsense word of length greater than 10
F₁₀	domain_length	Numerical (integer)	Length of the domain
F₁₁	url_length	Numerical (integer)	Total length of the URL
F₁₂	url_contains_ip	Boolean (0/1)	Presence of an IP address in the URL
F₁₃	presence_of_url_shortner	Boolean (0/1)	Presence of a URL shortening service in domain
F₁₄	special_characters_count	Numerical (count-based)	Total number of special characters in the URL
F₁₅	no_of_at	Numerical (count-based)	Count of ‘@’ symbols in the URL
F₁₆	no_of_comma	Numerical (count-based)	Count of ‘,’ symbols in the URL
F₁₇	no_of_dollar	Numerical (count-based)	Count of ‘$’ symbols in the URL
F₁₈	no_of_semicolon	Numerical (count-based)	Count of ‘;’ symbols in the URL
F₁₉	no_of_space	Numerical (count-based)	Count of spaces in the URL
F₂₀	no_of_ampersand	Numerical (count-based)	Count of ‘&’ symbols in the URL
F₂₁	no_of_dots	Numerical (count-based)	Count of dots in the URL
F₂₂	no_of_equal	Numerical (count-based)	Count of ‘=’ signs in the URL
F₂₃	no_of_percent	Numerical (count-based)	Count of ‘%’ characters in the URL
F₂₄	no_of_underscore	Numerical (count-based)	Count of ‘_’ symbols in the URL
F₂₅	no_of_hyphen	Numerical (count-based)	Count of ‘-’ characters in the URL
F₂₆	no_of_questionmark	Numerical (count-based)	Count of ‘?’ symbols in the URL
F₂₇	no_of_colon	Numerical (count-based)	Count of ‘:’ characters in the URL
F₂₈	no_of_slashes_inpath	Numerical (count-based)	Count of ‘/’ characters in the URL path
F₂₉	has_asterisk	Boolean (0/1)	Presence of ‘*’ in the URL
F₃₀	has_or	Boolean (0/1)	Presence of ‘’|’ in the URL
F₃₁	has_embedded_url	Boolean (0/1)	Presence of hidden URL in query
F₃₂	uses_https	Boolean (0/1)	Indicates whether the URL uses HTTPS scheme
F₃₃	num_query_params	Numerical (count-based)	Number of URL query parameters
F₃₄	numeric_proportion	Numerical (real-valued)	Proportion of digits in the URL
F₃₅	url_entropy	Numerical (real-valued)	Shannon entropy of the URL
F₃₆	hostname_digit_ratio	Numerical (real-valued)	Ratio of digits in the hostname
F₃₇	has_redirections	Boolean (0/1)	Additional occurrence of ‘//’ in URL beyond http:// or https://
F₃₈	has_punycode	Boolean (0/1)	Presence of Punycode in the URL
F₃₉	count_www	Numerical (count-based)	Count of ‘www’ occurrences in the URL
F₄₀	count_com	Numerical (count-based)	Count of ‘com’ occurrences in the URL
F₄₁	url_has_high_risk_extension	Boolean (0/1)	Presence of high-risk file extensions (e.g., .exe, .msi, etc.)
F₄₂	has_explicit_port	Boolean (0/1)	Indicates whether an explicit port is specified
F₄₃	total_words_url	Numerical (count-based)	Total number of words in the URL
F₄₄	average_length_of_words	Numerical (real-valued)	Average length of words in the URL
F₄₅	longest_word_length	Numerical (integer)	Length of the longest word in the URL
F₄₆	shortest_word_length	Numerical (integer)	Length of the shortest word in the URL
F₄₇	presence_of_free_hosting	Boolean (0/1)	Presence of a known free hosting domain

**3. Content-based Features**: These features are extracted from the HTML source code of a webpage. They focus on the content of the webpage rather than the URL structure and are critical in detecting the presence of malicious hyperlinks, JavaScripts, and auto-downloadable files. Additionally, they also include attributes capable of identifying compromised legitimate domains.

Table 4 presents a description of these features.

**Table 4:** Feature description: content-based features.

S. No	Feature	Type of data	Description
F₄₈	no_of_forms	Numerical (integer)	Number of form elements on the webpage
F₄₉	no_of_images	Numerical (integer)	Total number of images in the webpage
F₅₀	no_of_hyperlinks	Numerical (integer)	Total number of hyperlinks in the webpage
F₅₁	no_of_external_links	Numerical (integer)	Hyperlinks redirecting to external domains
F₅₂	no_of_internal_links	Numerical (integer)	Hyperlinks directing to the same domain
F₅₃	no_of_null_links	Numerical (integer)	Hyperlinks with no valid target
F₅₄	no_of_selfreference_links	Numerical (integer)	Links pointing to the same resource
F₅₅	no_of_empty_links	Numerical (integer)	Links with href attribute as empty or ‘#’
F₅₆	EmptyToExternalLinksRatio	Numerical (real-valued)	Ratio between empty and external links
F₅₇	Risky_extensions_In_Html	Boolean (0/1)	Presence of suspicious file extensions in HTML content (e.g., .exe, .msi, etc.)
F₅₈	presence_of_shorten_url_html	Boolean (0/1)	Presence of URL shortening service in the HTML code
F₅₉	hidden_elements_html	Boolean (0/1)	Presence of hidden elements in the webpage
F₆₀	Script_loaded_from_ext_domain	Boolean (0/1)	Presence of JavaScript loaded from external domains
F₆₁	suspicious_inline_js	Boolean (0/1)	Presence of suspicious inline JavaScript
F₆₂	is_footer_mismatch	Boolean (0/1)	Footer content mismatch with the domain
F₆₃	is_header_mismatch	Boolean (0/1)	Header content mismatch with the domain
F₆₄	is_copyright_mismatch	Boolean (0/1)	Copyright information mismatch with the actual brand
F₆₅	is_socials_mismatch	Boolean (0/1)	Social media links mismatch with the domain
F₆₆	is_favicon_mismatch	Boolean (0/1)	Favicon mismatch with the domain
F₆₇	has_missing_title	Boolean (0/1)	Webpage missing a title tag
F₆₈	title_mismatch_with_domain	Boolean (0/1)	Title mismatch with the domain name
F₆₉	has_inline_event_handlers	Boolean (0/1)	Presence of inline event handlers (e.g., onclick, onhover) in hyperlinks or buttons
F₇₀	form_action_external	Boolean (0/1)	Form action attribute submitting data to an external domain

The dataset comprises features having boolean values, count-based numerical features which capture occurrence frequencies, integer-valued numerical attributes that represent structural properties, and real-valued parameters reflecting the statistical measures*.*

To provide additional insights into the dataset characteristics, feature distribution statistics are provided. Table 5, Table 6 summarize the statistics for representative numerical and binary features, respectively. The numerical features exhibit substantial variability, reflecting the diversity of the dataset in terms of URL and webpage structure. The binary features occur less frequently as they capture specific impersonation techniques rather than general phishing characteristics.

**Table 5:** Summary statistics of representative numerical features.

Feature	Mean	Standard Deviation	Minimum	Maximum
url_length	40.06	66.91	13	6310
no_of_dots	1.97	0.82	1	16
no_of_slashes_inpath	1.23	0.82	0	22
special_characters_count	1.10	10.68	0	1270
no_of_hyperlinks	166.14	332.19	0	9488
no_of_external_links	59.75	184.51	0	9144
no_of_forms	0.97	3.20	0	200

**Table 6:** Prevalence of representative binary features.

Feature	Positive Count	Positive Percentage (%)
brand_typo_dom	137	0.89
brand_in_path_query	77	0.50
url_has_high_risk_extension	1401	9.12

**Fig. 2:**
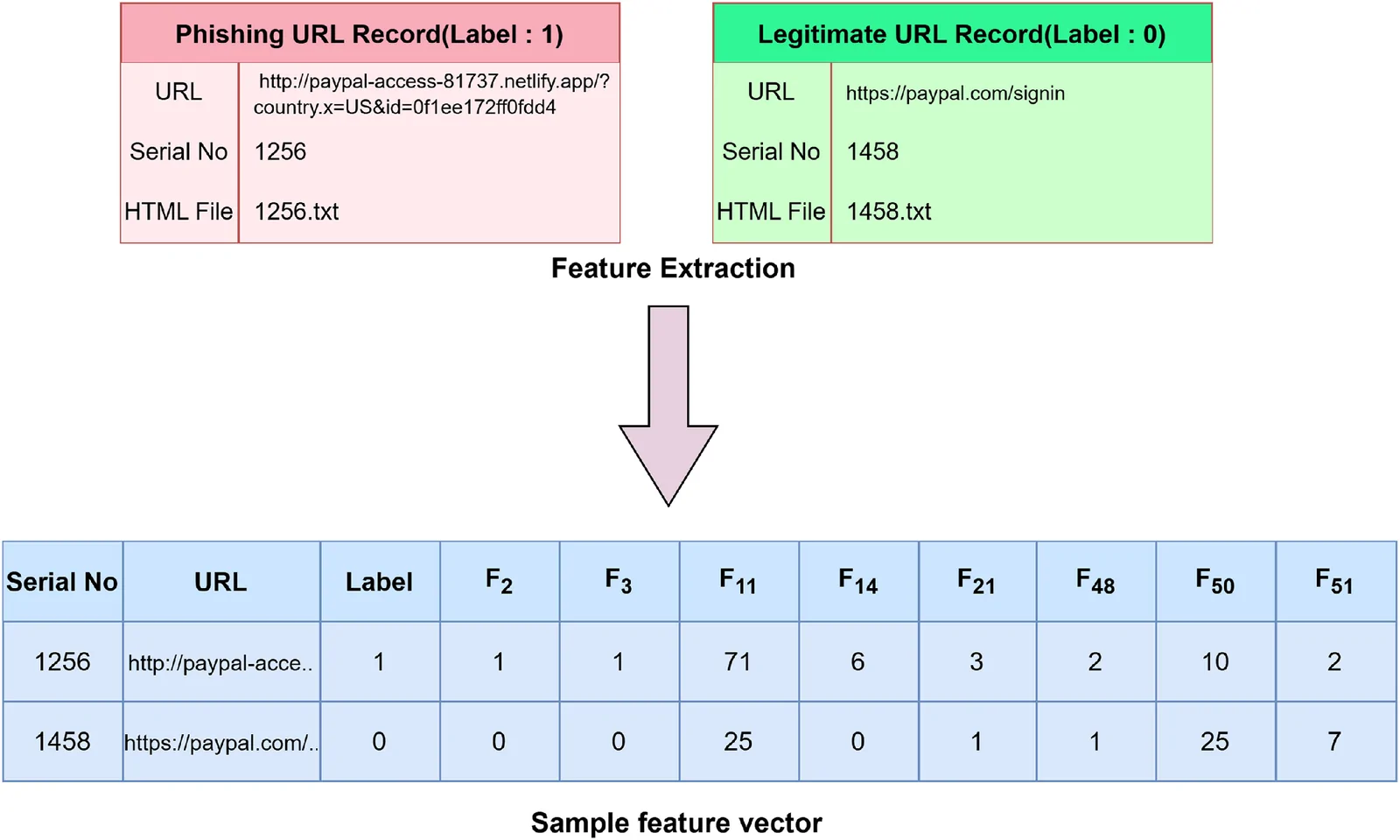
Sample dataset record and feature vector.

To facilitate understanding for future users, Fig. 2 presents sample dataset records and feature vectors. Each dataset record consists of a URL, its serial number, associated HTML source file, and a class label. Post feature extraction, these records are transformed into a set of 70 features (F_1_–F_70_) comprising brand-jacking, URL-based lexical/structural, and content-based attributes. For illustration purposes, only a subset of the extracted features is shown.

## 4 Experimental Design, Materials and Methods

This section presents a detailed description of the data acquisition process and the software and hardware resources employed. The process of extracting various categories of features is also discussed with the help of algorithms.

The raw samples of the dataset (i.e.*,* the URLs and the HTML source code) are collected over twelve months from September 2024 to August 2025. Data collection is performed incrementally to capture a diverse set of phishing campaigns and legitimate websites. The initial dataset consisted of approximately 5300 raw samples collected during the early stages of the study. Subsequently, additional phishing and legitimate URLs were gathered, and their corresponding HTML source code was archived. Through this continuous collection process, the dataset is expanded to a final size of 15,358 samples. To reduce potential class imbalance effects, phishing and legitimate URLs were collected in comparable proportions during dataset collection, with final dataset comprising 46.9% phishing and 53.1% legitimate samples. The extended collection period enabled the inclusion of phishing websites associated with different campaigns and time frames, thereby improving the diversity and representativeness of the dataset. Feature extraction followed thereafter.

Duplicate URLs, and those URLs that failed to return a webpage due to inactivity or a connection timeout, are discarded from the final dataset. Standard HTTP redirect handling is enabled to facilitate webpage retrieval. The final reachable webpage content is stored for subsequent analysis. Additionally, the presence of potential external redirection pattern (multiple occurrence of ‘//’ beyond the protocol delimiter i.e.*,* http:// or https://) is analyzed as a lexical URL feature, F_37_.

The entire process of dataset acquisition and feature extraction is performed on a 10th-generation Intel Core i5 computer with a 2.4 GHz processor using Python 3.x. Data collection and processing was implemented using standard Python libraries, including *Requests, Selenium, BeautifulSoup4, tldextract, Levenshtein, TheFuzz, Pandas*, and *NumPy*. The experiments were conducted on 64-bit Windows operating system with 8 GB RAM. Python standard library modules, including *urllib.parse, pickle, logging, csv, re, os*, and *concurrent.futures*, were used for URL parsing, loading the pre-trained gibberish detection model [14], execution logging, data processing, regular expression matching, file handling, and multithreaded webpage retrieval, respectively. To facilitate reproducibility, the complete list of software dependencies and their exact versions is provided in the *requirements.txt* file included with the dataset repository.

• **Phishing URLs acquisition**: Active phishing URLs are collected from *PhishTank* [15] and *OpenPhish* [16] repositories.
• **Legitimate URLs acquisition**: Multiple independent sources, such as *GitHub* [17,18], *DataforSEO* [19], personal blogs, business sites [20] are utilized to collect legitimate URLs*.*
• **HTML source codes:** The HTML source codes for both phishing as well as legitimate URLs are collected through a Python script. The initial list contains both URLs and domains. However, to download the HTML source code, the domains are needed to be resolved into valid URLs through HTTPS followed by HTTP connection. The first successfully resolved URL is retained for subsequent HTML acquisition. The HTML source code files are released in their original form to preserve the structural and behavioural characteristics necessary for phishing detection research and may contain potentially malicious scripts. Hence, the users are advised to operate under a controlled environment. To reduce the risk of accidental execution, the source code is saved as plain-text (*.txt) files rather than executable (.html files).* Algorithm 1 depicts the entire process of downloading the HTML source codes and their mapping with the corresponding URLs.

**Table:** 

**Algorithm 1:** HTML files acquisition and Dataset Mapping.

Input: Excel file (*All_URLs.xlsx*) containing manually assigned list of Serial_Numbers (S_i_), URLs/Domains (U), and Labels (*L*_i_) where *L_i_* = 1 denotes phishing and *L_i_* = 0 denotes legitimate Output: Locally saved HTML source code, *D* = *D_phish_ D_legit_* and Curated mapping file (*Mapping_file.xlsx*) 1. Read the input Excel file (*All_URLs.xlsx*) into dataframe *M* 2. For each row (*S_i_, U_i_, L_i_*) in *M* do If *U_i_* is already a valid URL then Retain it without modification else Resolve the domain into a URL by attempting HTTPS followed by HTTP connections Retain the first successfully resolved URL end Store the validated *U_i_*, together with *S_i_* and *L_i_* in an intermediate Excel file (*All_URLs_New.xlsx*) end 3. Read *All_URLs_New.xlsx* into dataframe *M* 4. For each row (*S_i_, U_i_, L_i_*) in *M* do Attempt to retrieve the HTML source code using the Requests library if Response contains valid static HTML then Save HTML as *S_i_*.txt in *All_HTML* folder else Initialize Selenium WebDriver in headless mode Load *U_i_* and capture rendered HTML content if Rendered HTML retrieved successfully then Save HTML as *S_i_*.txt in *All_HTML* folder end end end 5. Generate *Mapping_file.xlsx* with only those (*S_i_, U_i_, L_i_*) whose *S_i_*.txt was successfully stored in *All_HTML* 6. Construct Dataset: *D* = *D_phish_* U *D_legit_* 7. *D* = *D_phish_* ← HTML files where *L_i_* = 1, *D_legit_ t* ← HTML files where *L_i_* = 0 *8.* return *D* and *Mapping_file.xlsx*
• **Brand-Jacking-based features**: These binary features are introduced in one of our earlier works [21], where a layered approach is employed to detect brand-jacking and lexical feature-based phishing. The value of these features depends on the presence of misspelled brand names of the most targeted brands in various components of the URL. Approximate string matching functions of the *fuzz library* of Python programming language are used with a similarity threshold of 90%.

A lower value of similarity threshold might increase the number of false positives, and a higher value might lead to a scenario where a brand-jacking case is left undetected. Algorithm 2 describes the conditions under which the values of these features are assigned as 1 or 0.

• **URL-based lexical/structural features:** These features are directly extracted from the URL and constitute numerical (integer, count-based, as well as real-valued) and boolean type of features. Their process of extraction is described in Algorithm 3.

**Table:** 

**Algorithm 3:** URL-based Lexical and Structural Feature Extraction (*F*_6_–*F*_47_).

Input: Excel file *Mapping_file.xlsx* containing Serial_Number (*S_i_*), URL (*U_i_*), and Label (*L_i_*) Output: Feature vector *F* (*U_i_*) = *F*_6_–*F*_47_ for each URL *U_i_* For each row (*S_i_, U_i_, L_i_*) in *Mapping_file.xlsx* do Parse URL *U_i_* into components: *protocol, domain, subdomain, path, query, and TLD* Extract lexical and structural features Concatenate the extracted feature values (*F*_6_–*F*_47_) with Excel file *All_features_threshold90.xlsx* end return Updated processed dataset containing lexical and structural features
• **Content-based features**: These features are extracted from the HTML source code corresponding to each URL. Algorithm 4 describes the process of extraction of these features listed in Table 4.

**Table:** 

**Algorithm 4:** Content-based feature extraction (*F*_48_–*F*_70_).

Input: (i)Excel file *Mapping_file.xlsx* containing Serial_Number (*S_i_*), URL (*U_i_*), and Label (*L_i_*) (ii) Corpus of locally stored HTML source code files, where each file *S_i_.txt* corresponds to *U_i_* Output: Feature vector *F* (*U_i_*) = *F*_48_–*F*_70_ for each URL *U_i_* For each row (*S_i_, U_i_, L_i_*) in *Mapping_file.xlsx* do Fetch the corresponding HTML source file *S_i_.txt* from local storage Parse HTML content using BeautifulSoup Extract content-based features from the HTML source Concatenate extracted feature values (*F*_48_–*F*_70_) with Excel file *All_features_threshold90.xlsx* end return Updated processed dataset containing content-based features

**Table:** **Algorithm 2:** Brand-Jacking-based feature extraction(*F*_1_–*F*_5_).

1. Input: Excel file *Mapping_file.xlsx* containing serial number *Sᵢ*, URL *U_i i_*, label *Lᵢ*, brand list *B = {b₁, b*₂, *…, b_m_}* (brand length ≥ 6), and Protected Domain list *P*
Output: Feature vector *F(U_i_)* = [F₁, F₂, F₃, F₄, F₅] for each URL *U_i_*
2. For each URL *U_i_* in *Mapping_file.xlsx* do
Parse *U_i_* to extract components: *hostname, subdomain, domain, top-level domain (TLD), path*, and *query*.
3. For each brand *bⱼ* ∈ B do
3.1 If *U_i_* corresponds to a legitimate or protected domain of *b*ⱼ (i.e., *domain* ∈ *P[bⱼ])* then
Continue // Skip legitimate domains
end
3.2 If any of the following cases occur in *subdomain* then
Set F_1_ ← 1. // Brand in subdomain
a) Brand name as subdomain (e.g., *amazon.nbhqy.com*)
b) Brand as substring in subdomain (e.g., *facebookqjky.vmlx98.com*)
c) Misspelled brand in subdomain (e.g., *amazom.shoppin.com*)
d) Brand with separator (‘-’, ‘_’, ‘.’) (e.g., *amazon-sdwxz.shop.com*)
e) Misspelled brand with separator (e.g., *amazcon-bfgdfd.hobby.com*)
f) Brand infused with separator (e.g., *swiss-passv6.web.app*)
g) Misspelled brand infused with separator (e.g., *face-bo0k.phish.com*)
h) Misspelled brand as substring (e.g., *facebo0klog.phish.com*)
end
3.3 If *bⱼ* occurs as a substring in *domain* then
Set F_2_ ← 1. // Brand substring in domain
end
3.4 If any of the following cases occur in *domain* then
Set F_3_ ← 1. // Brand typo in domain
a) Misspelled brand (e.g., *amaz0nn.com*)
b) Brand with separator (‘-’, ‘_’) (e.g., *sav-netflix-infos.com*)
c) Misspelled brand with separator (e.g., *psypay-basnk.com*)
d) Brand infused with separator (e.g., *yahoo-mail.com*)
e) Misspelled brand infused with separator (e.g., *meta-masken.com*)
end
3.5 If domain = domain(*bⱼ*) and TLD ∉ TLD(*bⱼ*) then
Set F_4_ ← 1. // Fake top-level domain
End
3.6 If approximate string matching detects *bⱼ* in *path* or *query* then
Set F_5_← 1. // Brand in path or query
end
4. Store feature vector F(*U_i_*) = [F₁, F₂, F₃, F₄, F₅] in *All_features_threshold90.xlsx*.
5. Return the updated processed dataset containing Brand-Jacking-based features.

## Limitations

Periodic updates may be required to maintain the relevance of the dataset. As phishing attacks evolve and attackers target newer brands, the brand list used for brand-jacking detection may need to be expanded. Furthermore, despite incorporating raw samples from diverse sources, the use of public repositories may introduce source-specific biases, and the dataset may not fully represent all geographic, linguistic, or emerging phishing variations. Phishing websites are short-lived and may become inactive after being reported. As a result, some URLs in the dataset may no longer be accessible to future users, although their corresponding HTML source codes have been archived. Additionally, the dataset reflects phishing characteristics observed during the specified data-collection period. Hence, changes in phishing attack strategies over time may result in emerging techniques that are not represented in the current dataset. These factors should be considered when using the dataset for model training, evaluation, and benchmarking.

## CRediT Author Statement

**Richa Goenka:** Conceptualization, Methodology, Formal analysis, Data curation, writing original draft**. Meenu Chawla**: Project administration, Investigation, Validation, Visualization, Supervision. **Namita Tiwari:** Supervision, Investigation, Validation, Visualization.

## Ethics Statement

The authors have read and followed the ethical requirements for publication in Data in Brief and confirm that the current work does not involve human subjects, animal experiments, or any data collected from social media platforms. The dataset contains archived HTML source code collected from live phishing websites. To preserve the authenticity of webpage characteristics, the HTML content is released in its original form and was not sanitized prior to publication. All HTML source codes are stored as plain-text (*.txt) files rather than executable (*.html) files. The dataset is intended solely for cybersecurity research and educational purposes, and researchers are advised to handle the files within isolated or controlled environments.

## Data Availability

Mendeley DataCompPhish (Original data).
